# Supplementary material for: Phenobarbital Induces SLC13A5 Expression through Activation of PXR but Not CAR in Human Primary Hepatocytes
Source: Cells. 2021 Dec 1;10(12):3381. doi: 10.3390/cells10123381 (PMC8699749; doi:10.3390/cells10123381)
Supplement: Supplementary file 1 [file cells-10-03381-s001.zip › cells-1487905-supplementary.pdf]

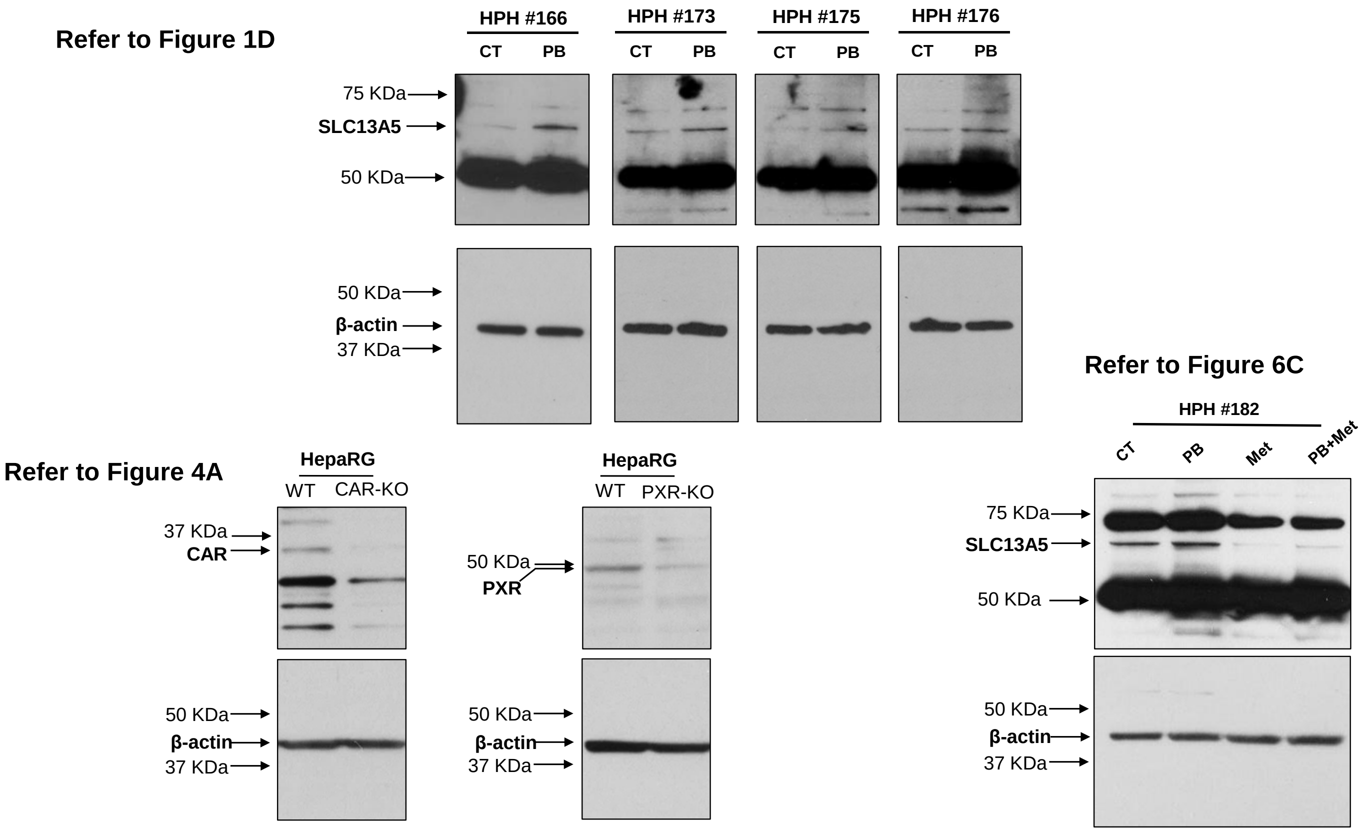

Figure S1. Complete images of the Western blotting. Complete images of the Western blots are presented referring to Figures 1D, 4A, and 6C of the manuscript.

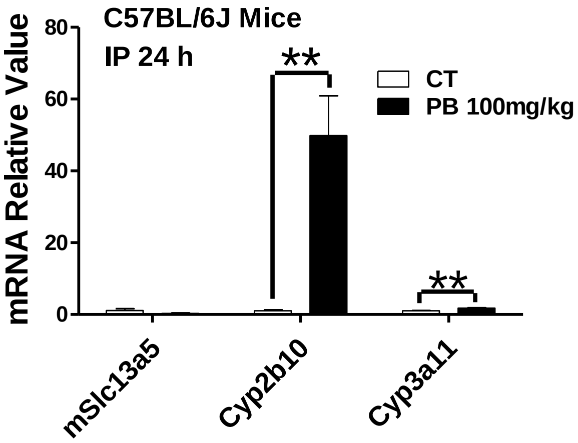

Figure S2. PB treatment does not affect mouse Slc13a5 expression. C57BL/6J mice were treated (IP injection) with vehicle control (CT) or PB (100 mg/kg) for 24 h. Real-time PCR was used to measure the relative mRNA abundance of Slc13a5, Cyp2b10 and Cyp3a11 genes. Data are expressed as the mean ± S.D. (*n* = 3 for animal study). ** $p < 0.01$.
